# Histological grading of tertiary lymphoid structures correlates with renal impairment and adverse outcomes in ANCA-associated glomerulonephritis

**DOI:** 10.3389/fimmu.2026.1916074

**Published:** 2026-09-16

**Authors:** Yujia Wang, Kaiqi Huang, Jiaye Fang, Caiming Chen, Zigui Zheng, Xuan Tao, Jianxin Wan, Hong Chen, Huabin Ma, Yanfang Xu

**Affiliations:** 1 Department of Nephrology, Blood Purification Research Center, the First Affiliated Hospital, Fujian Medical University, Fuzhou, Fujian, China; 2 Research Center for Metabolic Chronic Kidney Disease, the First Affiliated Hospital, Fujian Medical University, Fuzhou, Fujian, China; 3 Department of Nephrology, National Regional Medical Center, Binhai Campus of the First Affiliated Hospital, Fujian Medical University, Fuzhou, Fujian, China; 4 Department of Pathology, the First Affiliated Hospital, Fujian Medical University, Fuzhou, Fujian, China; 5 Central Laboratory, the First Affiliated Hospital, Fujian Medical University, Fuzhou, China

**Keywords:** ANCA-associated glomerulonephritis, histological grading, immune infiltration, renal prognosis, tertiary lymphoid structures

## Abstract

**Background:**

Tertiary lymphoid structures (TLS) are organized immune aggregates that develop in non-lymphoid tissues during chronic inflammation and are associated with adverse outcomes in various kidney diseases. Their role in antineutrophil cytoplasmic antibody (ANCA)-associated glomerulonephritis remains poorly characterized.

**Methods:**

This single-center retrospective study included 74 patients with biopsy-confirmed ANCA-associated glomerulonephritis. Intrarenal TLS were graded from G0 to G4 and dichotomized into low-grade (G0-2, n=37) and high-grade (G3-4, n=37). Clinical, laboratory, histopathological, and immunohistochemical parameters, systemic inflammatory indices, and progression to end-stage renal disease (ESRD) were compared between groups. Renal survival was analyzed using Kaplan-Meier and univariate and multivariate Cox regression to identify independent factors independently associated with ESRD. A prognostic nomogram incorporating these independently associated factors was constructed and internally validated using bootstrap-resampled calibration curves and the C-index.

**Results:**

High-grade TLS patients exhibited significantly lower estimated glomerular filtration rate (9.3 vs. 21.4 mL/min/1.73 m², p<0.001), higher Birmingham Vasculitis Activity Score (17.0 vs. 14.0, p=0.016), lower complement C3, and higher C-reactive protein, as well as a greater prevalence of crescentic/sclerotic histology (89.2% vs. 21.6%, p<0.001). High-grade TLS biopsies showed increased CD20+ B-cell and CD21+ follicular dendritic cell infiltration, while peripheral blood displayed a higher percentage of CD3+ T cells and an elevated Inflammation Burden Index (IBI). ESRD occurred in 51.4% of high-grade versus 27.0% of low-grade patients (p=0.032). High-grade TLS was associated with worse renal survival (log-rank p=0.021). In multivariate Cox regression, high-grade TLS, lower eGFR, lower complement C3, and sclerotic Berden class were identified as potential independent risk factors for ESRD in this cohort. A nomogram integrating these four predictors showed promising discriminative ability (C-index=0.897) and satisfactory calibration for predicting the probability of ESRD occurrence at 1 year.

**Conclusions:**

High-grade intrarenal TLS appear to be associated with more severe renal impairment, aggressive histopathology, and poor kidney survival in this ANCA-associated glomerulonephritis cohort. The finding that high-grade TLS may be independently associated with ESRD, together with the exploratory TLS-integrated nomogram, suggests a potential role for TLS grading in risk stratification. However, these preliminary findings warrant validation in larger prospective cohorts before any clinical application can be recommended.

## 1 Introduction

Antineutrophil cytoplasmic antibody (ANCA)-associated vasculitis frequently involves the kidney, with ANCA-associated glomerulonephritis representing the most severe organ manifestation and a major determinant of long-term outcome (1). The 2010 Berden histopathological classification stratifies renal lesions into focal, crescentic, mixed, and sclerotic classes based on predominant glomerular injury patterns (2). While subsequent validation studies confirm its prognostic value-with best renal survival in focal class and poorest in sclerotic class-substantial outcome heterogeneity persists even after integrating clinical parameters such as baseline estimated glomerular filtration rate into refined risk scores (2, 3). Experimental evidence suggests that the same ANCAs can elicit divergent patterns and severities of tissue injury depending on the local inflammatory milieu (4), raising the possibility that aspects of the local intrarenal immune microenvironment that are not fully captured by current classification schemes may also influence disease progression, and that more detailed characterization of these features could offer additional prognostic insights.

Tertiary lymphoid structures (TLS), also known as ectopic lymphoid tissues (ELT), are organized, non-encapsulated aggregates of immune cells that develop in non-lymphoid tissues in response to chronic inflammatory stimuli, including autoimmunity, infection, and ageing (5). Unlike secondary lymphoid organs, TLS lack a discrete capsule and are directly exposed to the local microenvironment, enabling them to function as *in situ* hubs for adaptive immunity (6). The presence and maturation stage of TLS have emerged as important modulators of disease course across a spectrum of chronic conditions: in cancer, TLS generally correlate with favorable outcomes and enhanced response to immunotherapy, whereas in autoimmunity and age-related inflammatory disorders, they are typically associated with more aggressive phenotypes and poorer prognosis (5, 7). In the kidney, TLS have been identified in a range of chronic kidney diseases, including IgA nephropathy, lupus nephritis and membranous nephropathy. Notably, clinical studies have consistently demonstrated that the presence of intrarenal TLS is associated with worse kidney function, greater histopathological severity, and poorer long-term renal outcomes in these conditions (7). In a large multicenter cohort of patients with IgA nephropathy, a higher density of intrarenal TLS was identified as an independent risk factor for disease progression, particularly among those with preserved kidney function at baseline, suggesting a pathogenic role in the early stages of renal injury (8).

However, the role of TLS in ANCA-associated glomerulonephritis remains poorly defined. To explore this relationship, we stratified a cohort of patients with ANCA-associated glomerulonephritis according to the degree of TLS organization observed in renal biopsies and compared clinical, histopathological, and immunologic characteristics between those with low-grade and high-grade TLS. The analysis aimed primarily to identify potential associations between TLS maturation and local immune infiltration, systemic inflammatory burden, and renal prognosis. Furthermore, we aimed to assess whether TLS grade was independently associated with ESRD and to construct an exploratory nomogram for individualized risk prediction.

## 2 Methods

### 2.1 Study design and patients

This retrospective observational study included patients who underwent percutaneous renal biopsy at the First Affiliated Hospital of Fujian Medical University between November 2019 and February 2026 and were diagnosed with ANCA-associated glomerulonephritis. Patients were eligible for inclusion if they had a confirmed diagnosis of ANCA-associated vasculitis with renal involvement, characterized by positive serology for myeloperoxidase (MPO)-ANCA or proteinase 3 (PR3)-ANCA and histopathological evidence of pauci-immune necrotizing or crescentic glomerulonephritis on kidney biopsy. Exclusion criteria included age under 18 years, overlap syndromes (e.g., concurrent anti-glomerular basement membrane disease or systemic lupus erythematosus), inadequate biopsy specimens, and incomplete baseline clinical or follow-up data. The study was approved by the Institutional Ethics Committee of the First Affiliated Hospital of Fujian Medical University (protocol code: [2026]-181) and was conducted in accordance with the Declaration of Helsinki. Written informed consent was obtained from all participants or their legal representatives.

### 2.2 Clinical and laboratory data collection

Demographic, clinical and laboratory data were retrospectively collected from the electronic medical record system. Demographic variables included age and sex. Comorbidities recorded were hypertension and diabetes. ANCA serotype was classified as myeloperoxidase (MPO)-ANCA or proteinase 3 (PR3)-ANCA based on enzyme-linked immunosorbent assay results. Laboratory parameters obtained at the time of renal biopsy included hemoglobin, white blood cell count with differentials (neutrophil, lymphocyte, and monocyte counts), platelet count, peripheral blood lymphocyte subsets (the percentages and absolute counts of CD3-positive T cells, CD4-positive T cells, CD8-positive T cells, and CD19-positive B cells, measured by flow cytometry), serum creatinine, serum albumin, complement C3 (normal reference range at our institution: 0.76-1.70 g/L), C-reactive protein, and erythrocyte sedimentation rate. The estimated glomerular filtration rate (eGFR) was calculated using the CKD-EPI equation. Urinalysis parameters included urinary albumin-to-creatinine ratio and urinary red blood cell count. Disease activity was assessed using the Birmingham Vasculitis Activity Score (BVAS). Treatment data were extracted, including induction regimens with corticosteroids, intravenous cyclophosphamide, rituximab, and plasma exchange. Of note, avacopan was not available at our center during the study period, and thus no patients received this agent. Cumulative prednisone-equivalent dose over the first 3 months was calculated. All patients underwent renal biopsy at first diagnosis; no repeat biopsies were included. The median interval from symptom onset to biopsy was 50 [19, 121] days, and the median interval from biopsy to treatment initiation was 2 [-5, 5] days.

### 2.3 Histopathological evaluation and TLS grading

Renal biopsy specimens were processed for light microscopy, immunofluorescence, and electron microscopy according to standard institutional protocols. Histopathological classification of ANCA-associated glomerulonephritis was performed according to the Berden criteria, categorizing each case as focal, crescentic, mixed, or sclerotic class. The Berden classification was specifically re-assessed for this study by two experienced pathologists who were blinded to clinical outcomes and TLS grading.

Immunohistochemical staining was performed on formalin-fixed, paraffin-embedded renal biopsy sections using antibodies against the following immune cell markers: CD20 (B cells), CD21 (follicular dendritic cells), CD3 (pan-T cells), CD4 (T helper cells), CD8 (cytotoxic T cells), CD68 (pan-macrophages), and CD163 (M2-like macrophages). Staining was visualized using a horseradish peroxidase-conjugated secondary antibody system with 3,3’-diaminobenzidine (DAB) as the chromogen. The degree of immune cell infiltration for each marker was assessed semi-quantitatively by two independent observers blinded to clinical data and renal outcomes.

Tertiary lymphoid structures (TLS) were identified on hematoxylin and eosin (H&E)-stained sections as dense aggregates of lymphocytes in the tubulointerstitial compartment. TLS were further characterized by immunohistochemical staining for CD20 (B cells), CD3 (T cells), and CD21 (follicular dendritic cells). The degree of TLS organization was graded according to previously described criteria (9), ranging from G0 (absent or loose lymphocytic aggregates without CD21-positive follicular dendritic cell networks) to G4 (well-organized structures with distinct B-cell follicles, T-cell zones, and prominent CD21-positive follicular dendritic cell networks). For the purpose of this analysis, patients were stratified into two groups: low-grade TLS (G0–G2) and high-grade TLS (G3–G4).

### 2.4 Systemic inflammatory composite indices

Nine established systemic inflammatory composite indices were calculated from complete blood count and biochemical parameters obtained at baseline: the Systemic Immune-Inflammation Index (SII; platelet count × neutrophil count/lymphocyte count), the Systemic Inflammation Response Index (SIRI; neutrophil count × monocyte count/lymphocyte count), the Neutrophil-to-Lymphocyte Ratio (NLR), the Platelet-to-Lymphocyte Ratio (PLR), the Aggregate Index of Systemic Inflammation (AISI; neutrophil count × monocyte count × platelet count/lymphocyte count), the Monocyte-to-Lymphocyte Ratio (MLR), the Neutrophil-to-Platelet Ratio (NPR), the Pan-Immune-Inflammation Value (PIV; neutrophil count × platelet count × monocyte count/lymphocyte count), and the Inflammation Burden Index (IBI; CRP × neutrophil count/lymphocyte count).

### 2.5 Follow-up and outcome definition

Patients were followed from the date of diagnostic renal biopsy until the occurrence of end-stage renal disease (ESRD), death, loss to follow-up, or the last follow-up visit (February 2026), whichever came first. The primary outcome was the development of ESRD, defined as the need for long-term renal replacement therapy (maintenance dialysis or kidney transplantation) or a sustained eGFR below 15 mL/min/1.73 m² for at least three months. Renal survival time was calculated from the date of biopsy to the date of ESRD onset.

### 2.6 Statistical analysis

Categorical variables were expressed as frequencies and percentages and compared between groups using the chi-square test or Fisher’s exact test, as appropriate. Continuous variables were assessed for normality using the Shapiro-Wilk test. Normally distributed data were presented as mean ± standard deviation and compared using the independent samples t-test, whereas non-normally distributed data were expressed as median (interquartile range) and compared using the Mann-Whitney U test. Renal survival was analyzed using the Kaplan-Meier method, and differences between groups were assessed with the log-rank test. Univariate and multivariate Cox proportional hazards regression models were used to estimate hazard ratios (HRs) and 95% confidence intervals (CIs) for the association between TLS grade and ESRD. Variables with a p-value <0.10 in univariate analysis, along with clinically established risk factors, were entered into the multivariate model using a forward stepwise selection procedure to assess independent associations with ESRD. The final multivariate Cox model included 5 covariates (TLS grade, eGFR, complement C3, and Berden classification), with a total of 29 ESRD events, yielding an events-per-variable ratio of approximately 5.8. A prognostic nomogram was constructed based on the independent risk factors identified in the multivariate Cox model to predict the probability of ESRD occurrence at 1 year. The discriminative ability of the nomogram was evaluated using Harrell’s concordance index (C-index), and internal validation was performed using 1000 bootstrap resamples to generate calibration curves, which compared the predicted ESRD probability versus the observed ESRD outcomes. All statistical tests were two-sided, and a P value < 0.05 was considered statistically significant. Analyses were conducted using IBM SPSS Statistics (version 26.0), GraphPad Prism (version 8.0.2), and R software (version 4.5.2).

## 3 Results

### 3.1 Baseline characteristics

A total of 74 patients with ANCA-associated glomerulonephritis were divided into low-grade tertiary lymphoid structures (TLS, G0-2, n=37) and high-grade TLS (G3-4, n=37) groups according to the degree of TLS in renal biopsies. Baseline demographic, clinical, laboratory and histopathological characteristics are summarized in **Table 1**. Patients with high-grade TLS were significantly older (median 65.0 [57.0–69.0] vs. 58.0 [52.0–64.0] years, p=0.048) and presented with more severe renal impairment, as reflected by a markedly lower eGFR at admission (9.3 [6.1–16.9] vs. 21.4 [10.3–53.1] mL/min/1.73 m², p<0.001). Compared with the low-grade TLS group, patients with high-grade TLS exhibited a significantly greater systemic inflammatory burden and disease activity, evidenced by a higher Birmingham Vasculitis Activity Score (BVAS) (median 17.0 [13.5–26.5] vs. 14.0 [10.5–18.0], p=0.016), elevated C-reactive protein levels (15.7 [6.4–57.8] vs. 2.7 [0.8–41.8] mg/L, p=0.025), and lower complement C3 levels (0.7 [0.6–0.8] vs. 0.8 [0.7–1.0] g/L, p=0.029). Histopathological classification according to Berden showed that high-grade TLS was significantly associated with a more aggressive phenotype: the crescentic class was observed in 17 patients (45.9%) in the high-grade group vs. 5 patients (13.5%) in the low-grade group, and the sclerotic class in 16 patients (43.2%) vs. 3 patients (8.1%), while the focal class was more common in the low-grade group (24 patients, 64.9% vs. 3 patients, 8.1%; mixed class: 1 patient, 2.7% vs. 5 patients, 13.5%; overall p< 0.001). The proportion of patients who progressed to end-stage renal disease (ESRD) was significantly higher in the high-grade TLS group compared with the low-grade TLS group (19 patients, 51.4% vs. 10 patients, 27.0%; p=0.032). Several baseline characteristics were comparable between the two groups. Demographic features, comorbidities, and most routine laboratory parameters—including hemoglobin, white blood cell counts, platelet count, serum albumin, urine ACR, and urinary RBC—showed no significant differences. ANCA serotype distribution and median MPO-ANCA and PR3-ANCA titers were also similar. Likewise, treatment parameters, including cumulative corticosteroid dose and the use of cyclophosphamide, rituximab, or plasma exchange, did not differ significantly between groups.

**Table 1:** Baseline characteristics of ANCA-associated glomerulonephritis patients stratified by TLS grade.

Variable	TLS low (n=37)	TLS high (n=37)	p value
Age (years)	58.0 [52.0, 64.0]	65.0 [57.0, 69.0]	**0.048**
Male [n (%)]	21 (56.7%)	19 (51.3%)	0.816
ANCA serotype [n (%)]			0.754
MPA	30 (81.1%)	32 (86.4%)	
GPA	7 (18.9%)	5 (13.6%)	
MPO-ANCA titer (RU/mL)	134.6 [18.4, 200.0]	143.4 [27.8, 200.0]	0.470
PR3-ANCA titer (RU/mL)	2.0 [2.0, 11.6]	2.0 [2.0, 2.7]	0.325
Hypertension [n (%)]	5 (13.5%)	9 (24.3%)	0.374
Diabetes [n (%)]	20 (54.0%)	24 (64.8%)	0.478
Pulmonary manifestations [n (%)]	20 (54.5%)	23 (62.2%)	0.638
ENT manifestations [n (%)]	7 (18.9%)	10 (27.0%)	0.581
Hemoglobin (g/L)	96.0 [71.0, 118.0]	85.0 [74.0, 94.0]	0.062
White blood cell (×10^9^/L)	7.2 [5.7, 9.5]	6.7 [5.4, 9.6]	0.738
Neutrophil count (×10^9^/L)	5.3 [3.9, 7.8]	5.4 [3.8, 7.8]	0.970
Lymphocyte count (×10^9^/L)	1.1 [0.8, 1.8]	1.2 [0.7, 1.4]	0.393
Monocyte count (×10^9^/L)	0.4 [0.3, 0.6]	0.3 [0.2, 0.5]	0.144
Platelet count (×10^9^/L)	225.0 [169.0, 306.0]	225.0 [170.0, 288.0]	0.974
eGFR (mL/min/1.73m²)	21.4 [10.3, 53.1]	9.3 [6.1, 16.9]	**<0.001**
Serum albumin (g/L)	33.8 [28.1, 39.0]	30.9 [28.5, 35.8]	0.444
Urine ACR (mg/g)	773.8 [237.9, 1217.2]	840.5 [540.9, 2633.0]	0.177
Urinary RBC (/μL)	128.2 [34.0, 330.1]	166.7 [63.0, 522.6]	0.378
Complement C3 (g/L)	0.8 [0.7, 1.0]	0.7 [0.6, 0.8]	**0.029**
C-reactive protein (mg/L)	2.7 [0.8, 41.8]	15.7 [6.4, 57.8]	**0.025**
Erythrocyte sedimentation rate (mm/h)	47.0 [28.0, 54.0]	61.5 [51.8, 69.2]	0.095
BVAS score	14.0 [10.5, 18.0]	17.0 [13.5, 26.5]	**0.016**
Berden histologic classification [n (%)]			<0.001
Focal	24 (64.9%)	3 (8.1%)	
Crescentic	5 (13.5%)	17 (45.9%)	
Sclerotic	3 (8.1%)	16 (43.2%)	
Mixed	5 (13.5%)	1 (2.7%)	
Cumulative corticosteroid dose over the first 3 months (mg)	980 [980.0, 1522.5]	1050 [980.0, 1741.3]	0.449
Intravenous CTX [n (%)]	13 (35.1%)	16 (43.2%)	0.634
Rituximab [n (%)]	14 (37.8%)	11 (29.7%)	0.624
Plasma exchange [n (%)]	6 (16.2%)	11 (29.7%)	0.269
End-stage renal disease (ESRD) [n (%)]	10 (27.0%)	19 (51.4%)	**0.032**

ANCA, Antineutrophil Cytoplasmic Antibody; TLS, Tertiary lymphoid structures; MPA, Microscopic Polyangiitis; GPA, Granulomatosis with Polyangiitis; MPO, Myeloperoxidase; PR3, Proteinase 3; ENT, Ear, Nose, and Throat; eGFR, estimated Glomerular Filtration Rate; ACR, Albumin-to-Creatinine Ratio; RBC, Red Blood Cell; BVAS, Birmingham Vasculitis Activity Score; CTX, Cyclophosphamide. Bold values indicate p < 0.05.

### 3.2 Histological features of TLS grades

Renal TLS were identified on hematoxylin and eosin-stained sections as dense aggregates of lymphocytes and further characterized by immunohistochemical staining for CD3 (pan-T cells), CD20 (B cells), and CD21 (follicular dendritic cells). Representative images of each TLS grade (G0-G4) are shown in **Figure 1**. Grade G0 exhibited no CD20^+^ B cells or CD3^+^ T cells, with no CD21^+^ follicular dendritic cell (fDC) networks. Grade G1 displayed scattered CD20^+^ B cells and CD3^+^ T cells, without CD21^+^ fDC networks. Grade G2 was characterized by CD3^+^ T cells and CD20^+^ B cells distributed in clusters without microanatomical separation, and no CD21^+^ fDC networks were observed. Grade G3 showed aggregation regions composed of CD3^+^ T cells and CD20^+^ B cells without a central fDC network. Grade G4 exhibited highly organized CD3^+^ T-cell and CD20^+^ B-cell zones with a central network of CD21^+^ fDCs.

**Figure 1:**
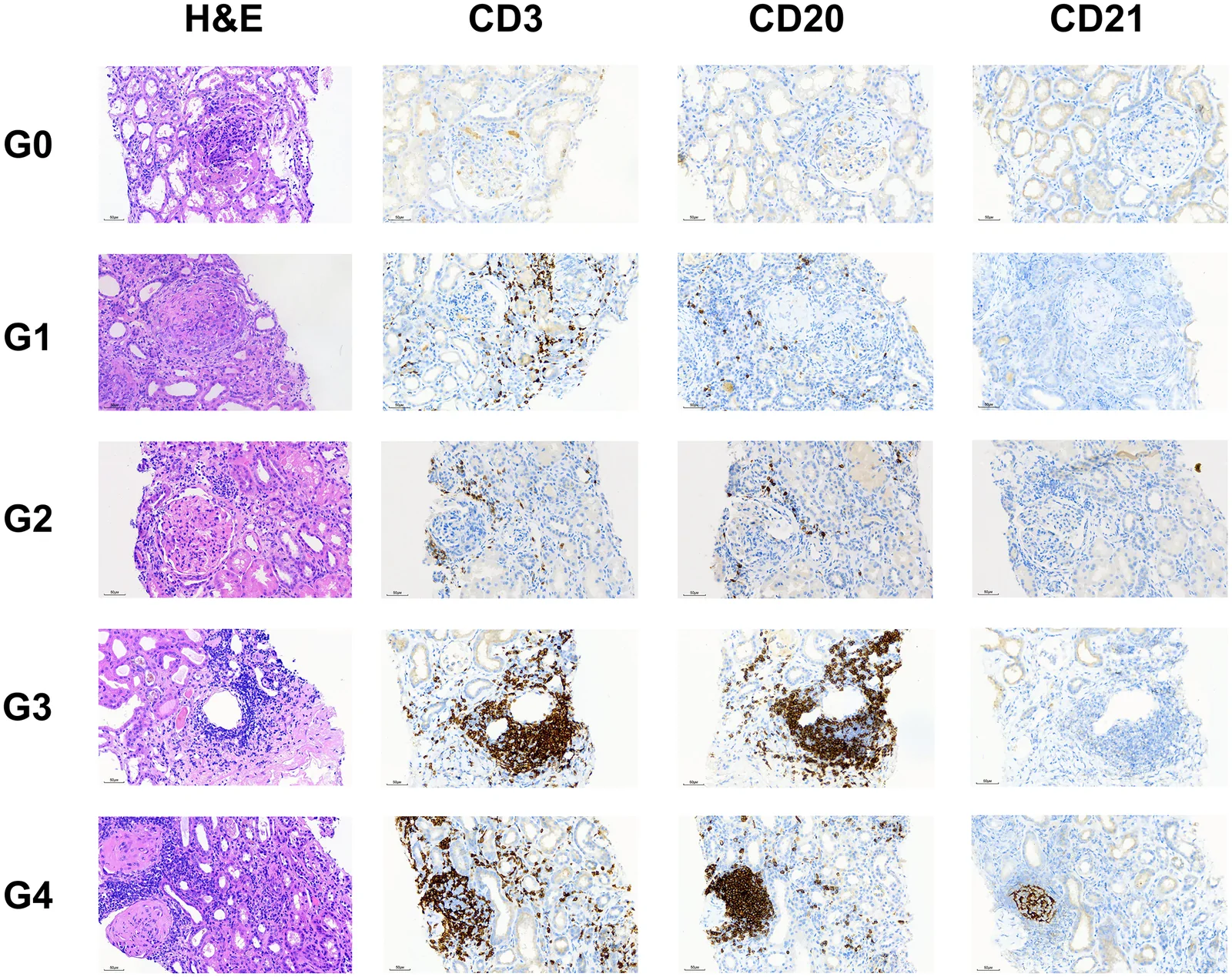
Representative histological images of TLS grading in ANCA-associated glomerulonephritis. Renal biopsy specimens from patients with ANCA-associated glomerulonephritis were stained with hematoxylin and eosin (HE) and immunostained for CD3 (pan-T cells), CD20 (B cells), and CD21 (follicular dendritic cells). Images are arranged by TLS grade from G0 to G4 (scale bar=50μm).

### 3.3 Renal immune cell infiltration

The infiltration grades of seven immune cell markers (CD20, CD21, CD3, CD4, CD8, CD68, CD163) in renal biopsies were compared between the two TLS groups (**Figure 2**). High-grade TLS was associated with significantly higher infiltration grades of CD20^+^ B cells (p=0.012) and CD21^+^ follicular dendritic cells (p<0.001). No significant differences were observed for CD3^+^ T cells, CD4^+^ T helper cells, CD8^+^ cytotoxic T cells, CD68^+^ macrophages, or CD163^+^ M2-like macrophages.

**Figure 2:**
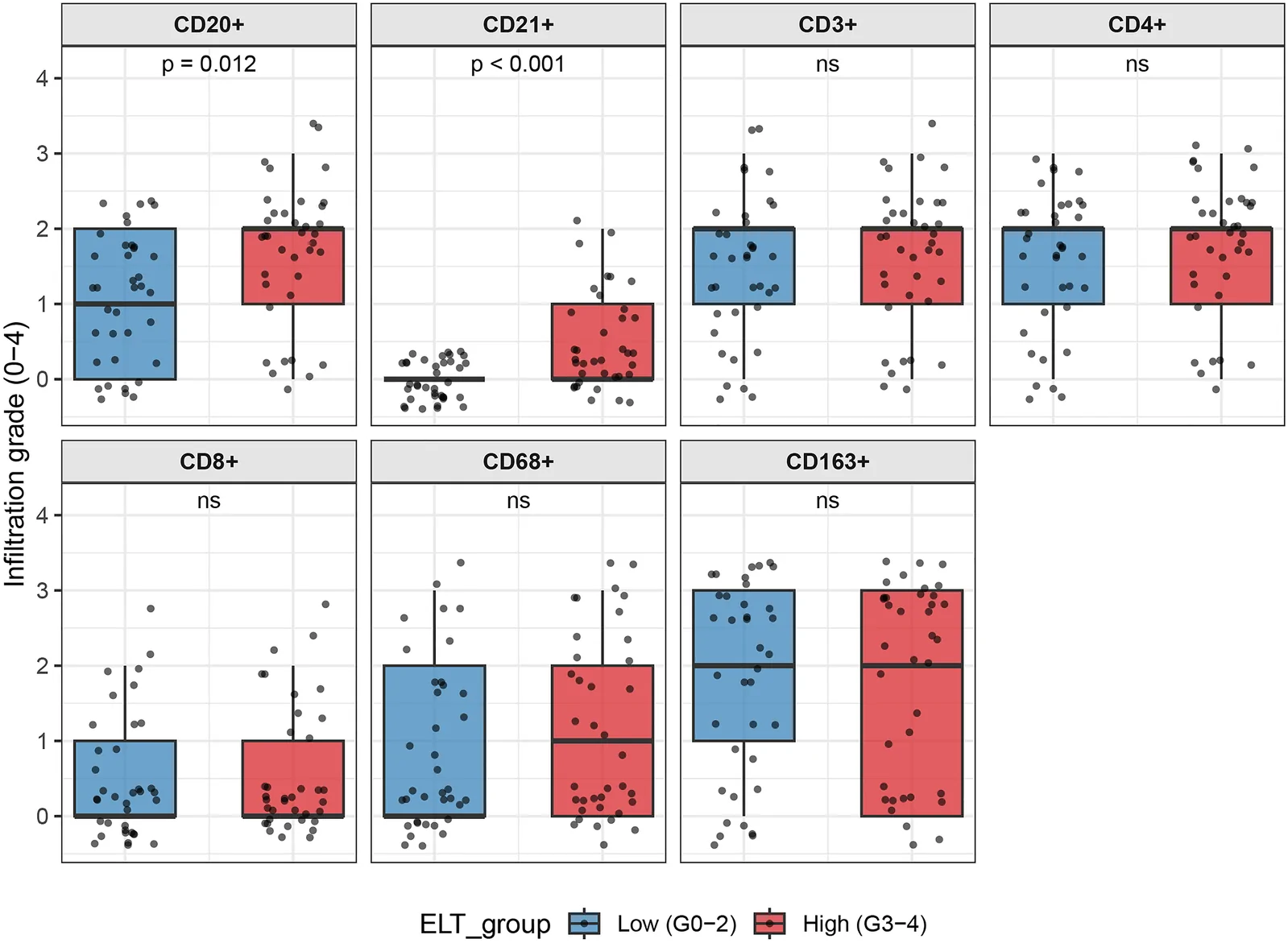
Renal immune cell infiltration according to TLS grade. Box plots showing the infiltration grades (0–4) of seven immune cell markers in renal biopsies from patients with low-grade (G0-2, n=37) and high-grade (G3-4, n=37) tertiary lymphoid structures (TLS). Markers include CD20+ B cells, CD21+ follicular dendritic cells, CD3+ T cells, CD4+ T helper cells, CD8+ cytotoxic T cells, CD68+ macrophages, and CD163+ M2-like macrophages. ns, not significant.

### 3.4 Peripheral blood lymphocyte subsets

Peripheral blood lymphocyte subsets were evaluated by flow cytometry (**Figure 3**). The percentage of CD3^+^ T cells was significantly higher in the high-grade TLS group compared with the low-grade TLS group (p=0.008). No significant differences were observed between the two groups in the counts of CD3^+^ T cells, the counts or percentages of CD4^+^ T cells, CD8^+^ T cells, the CD4/CD8 ratio, or CD19^+^ B cells.

**Figure 3:**
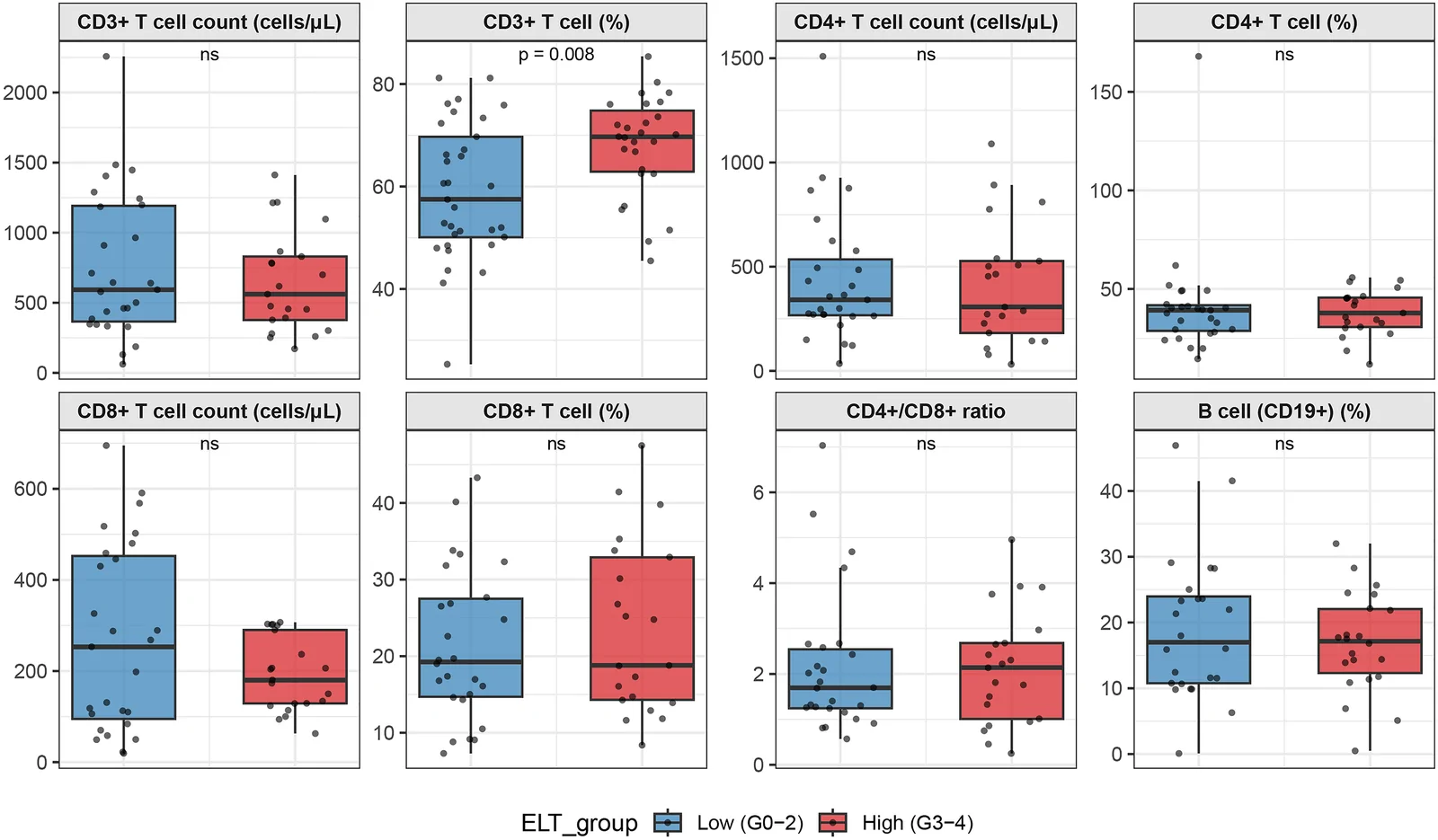
Peripheral blood lymphocyte subsets by TLS grade. Box plots showing the distribution of circulating lymphocyte populations in patients with low-grade (G0-2, n=37) and high-grade (G3-4, n=37) tertiary lymphoid structures (TLS). Parameters shown include CD3+ T cell percentage, CD3+ T cell count, CD4+ T cell count and percentage, CD8+ T cell count and percentage, CD4+/CD8+ ratio, and CD19+ B cell percentage. ns, not significant.

### 3.5 Systemic inflammatory composite indices

Nine established systemic inflammatory composite indices were analyzed (**Figure 4**). The Inflammation Burden Index (IBI) was significantly higher in the high-grade TLS group compared with the low-grade TLS group (p=0.025). No significant differences were observed between the two groups in the Systemic Immune-Inflammation Index (SII), Systemic Inflammation Response Index (SIRI), Neutrophil-to-Lymphocyte Ratio (NLR), Platelet-to-Lymphocyte Ratio (PLR), Aggregate Index of Systemic Inflammation (AISI), Monocyte-to-Lymphocyte Ratio (MLR), Neutrophil-to-Platelet Ratio (NPR), or Pan-Immune-Inflammation Value (PIV).

**Figure 4:**
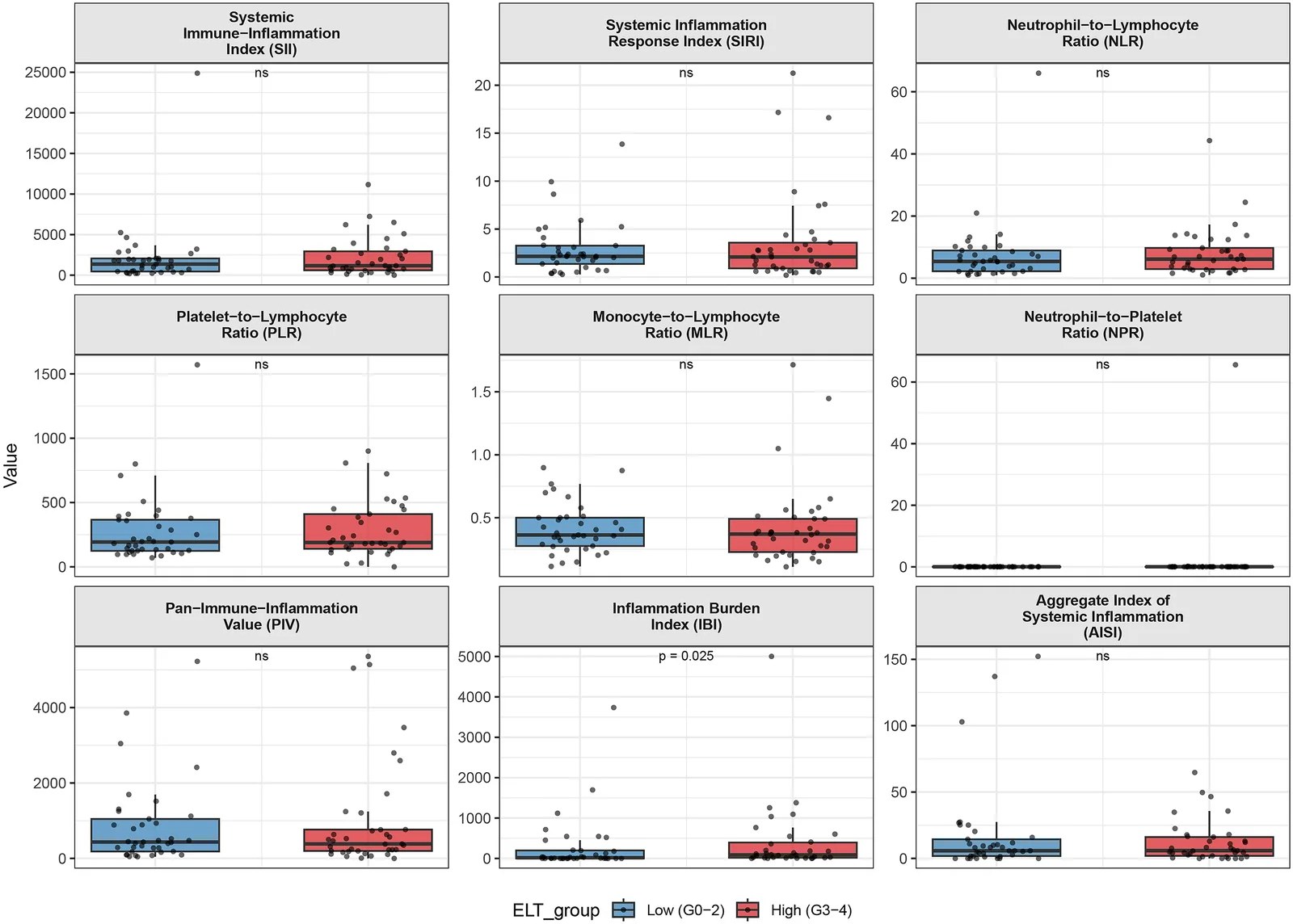
Systemic inflammatory composite indices according to TLS grade. Comparison of nine systemic inflammatory composite indices between low-grade (G0-2, n=37) and high-grade (G3-4, n=37) TLS groups. Indices include the Systemic Immune-Inflammation Index (SII), Systemic Inflammation Response Index (SIRI), Neutrophil-to-Lymphocyte Ratio (NLR), Platelet-to-Lymphocyte Ratio (PLR), Aggregate Index of Systemic Inflammation (AISI), Monocyte-to-Lymphocyte Ratio (MLR), Neutrophil-to-Platelet Ratio (NPR), Pan-Immune-Inflammation Value (PIV), and Inflammation Burden Index (IBI). ns, not significant.

### 3.6 Renal survival analysis and factors independently associated with ESRD

To further evaluate the association between TLS grade and renal prognosis, we performed Kaplan-Meier survival analysis with ESRD as the endpoint. The median follow-up time was 15.3 months. Patients with high-grade TLS had a significantly worse kidney survival compared with those with low-grade TLS (log-rank p=0.021; **Figure 5**). Univariate Cox regression analysis (**Table 2**) showed that high-grade TLS was associated with a significantly increased risk of ESRD (HR 2.189, 95% CI 1.016-4.714, p=0.045). Other univariate predictors included lower hemoglobin, lower eGFR, lower complement C3, and crescentic and sclerotic Berden classes and plasma exchange. In contrast, most demographic, comorbidity, extrarenal, and routine laboratory parameters showed no significant association with ESRD. Neither MPO-ANCA nor PR3-ANCA titers reached statistical significance. Among treatment-related factors, cumulative corticosteroid dose showed only a trend toward significance (p=0.057), while cyclophosphamide and rituximab were not significantly associated with ESRD. In the MPA subgroup sensitivity analysis, MPO-ANCA titers remained non-significant (**Supplementary Table 1**).

**Figure 5:**
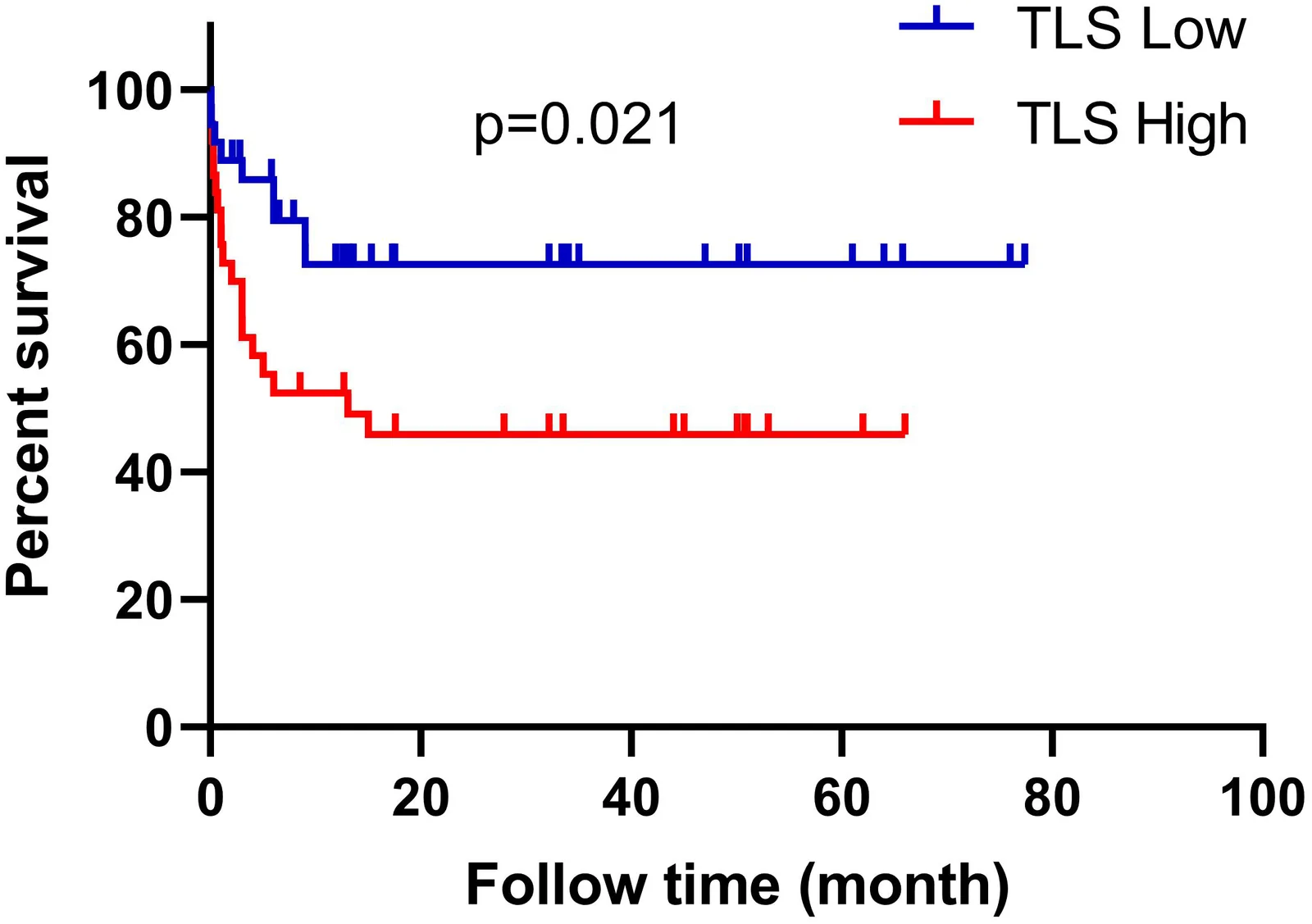
Renal survival according to TLS grade. Kaplan-Meier curves showing cumulative end-stage renal disease (ESRD)-free survival in patients with low-grade (G0-2, n=37) and high-grade (G3-4, n=37) TLS.

**Table 2:** Univariate Cox regression analysis of factors associated with end-stage renal disease (ESRD) in ANCA-associated glomerulonephritis patients.

Variable	HR (95% CI)	p value
Age (years)	1.015 [0.985, 1.046]	0.328
Male [n (%)]	0.815 [0.393, 1.689]	0.581
ANCA serotype [n (%)]		
MPA	Reference	
GPA	0.929 [0.323, 2.673]	0.892
MPO-ANCA titer (RU/mL)	0.996 [0.992, 1.001]	0.119
PR3-ANCA titer (RU/mL)	0.999 [0.991, 1.008]	0.845
Hypertension [n (%)]	1.533 [0.698, 3.367]	0.287
Diabetes [n (%)]	1.829 [0.807, 4.141]	0.148
Pulmonary manifestations [n (%)]	1.121 [0.533, 2.361]	0.763
ENT manifestations [n (%)]	1.033 [0.418, 2.553]	0.944
Hemoglobin (g/L)	0.969 [0.948, 0.991]	**0.005**
White blood cell (×10^9^/L)	0.995 [0.905, 1.094]	0.918
Neutrophil count (×10^9^/L)	1.013 [0.921, 1.115]	0.784
Lymphocyte count (×10^9^/L)	0.478 [0.227, 1.003]	0.051
Monocyte count (×10^9^/L)	0.310 [0.050, 1.937]	0.210
Platelet count (×10^9^/L)	1.000 [0.997, 1.003]	0.794
eGFR (mL/min/1.73m²)	0.877 [0.817, 0.941]	**<0.001**
Serum albumin (g/L)	0.936 [0.873, 1.004]	0.064
Urine ACR (mg/g)	1.000 [1.000, 1.000]	0.333
Urinary RBC (/μL)	1.000 [1.000, 1.001]	0.173
Complement C3 (g/L)	0.041 [0.008, 0.200]	**<0.001**
C-reactive protein (mg/L)	1.005 [0.996, 1.013]	0.290
Erythrocyte sedimentation rate (mm/h)	1.001 [0.976, 1.027]	0.933
BVAS score	1.028 [0.990, 1.068]	0.156
Berden histologic classification [n (%)]		
Focal	Reference	
Crescentic	6.042 [1.681, 21.714]	**0.006**
Sclerotic	10.632 [3.034, 37.189]	**<0.001**
Mixed	1.569 [0.163, 15.088]	0.696
Tertiary lymphoid structures (TLS) [n (%)]		
Low-grade TLS	Reference	
High-grade TLS	2.189 [1.016, 4.714]	**0.045**
Cumulative corticosteroid dose over the first 3 months (mg)	1.001 [1.000, 1.002]	0.057
Intravenous CTX [n (%)]	1.264 [0.607, 2.629]	0.531
Rituximab [n (%)]	0.447 [0.182, 1.099]	0.079
Plasma exchange [n (%)]	3.004 [1.407, 6.411]	**0.004**

ANCA, Antineutrophil Cytoplasmic Antibody; TLS, Tertiary lymphoid structures; MPA, Microscopic Polyangiitis; GPA, Granulomatosis with Polyangiitis; MPO, Myeloperoxidase; PR3, Proteinase 3; ENT, Ear, Nose, and Throat; eGFR, estimated Glomerular Filtration Rate; ACR, Albumin-to-Creatinine Ratio; RBC, Red Blood Cell; BVAS, Birmingham Vasculitis Activity Score; CTX, Cyclophosphamide. Bold values indicate p < 0.05.

To determine whether TLS grade provides independent prognostic information, we performed multivariate Cox regression analysis (**Table 3**). After adjusting for eGFR, complement C3, and Berden classification, high-grade TLS remained significantly and independently associated with ESRD (HR 4.625, 95% CI 1.575-13.582, p=0.005). Other independent risk factors included lower eGFR (HR 0.902, 95% CI 0.832-0.978, p=0.012), lower complement C3 (HR 0.027, 95% CI 0.004-0.181, p<0.001), and the sclerotic Berden class (HR 6.721, 95% CI 1.154-39.158, p=0.034). Plasma exchange was not retained as an independently associated factor in the multivariate model (p=0.581). To assess potential redundancy among these predictors, we performed collinearity diagnostics; variance inflation factor (VIF) values were 1.334 for TLS grade, 1.307 for Berden classification, 1.359 for eGFR, and 1.107 for complement C3, indicating no significant multicollinearity.

**Table 3:** Multivariate Cox regression analysis of factors independently associated with end-stage renal disease (ESRD) in ANCA-associated glomerulonephritis patients.

Variable	HR(95% CI)	p value
eGFR (mL/min/1.73m²)	0.902 [0.832, 0.978]	**0.012**
Complement C3 (g/L)	0.027 [0.004, 0.181]	**<0.001**
Berden histologic classification [n (%)]		
Focal	Reference	
Crescentic	2.589 [0.458, 14.623]	0.281
Sclerotic	6.721 [1.154, 39.158]	**0.034**
Mixed	0.547 [0.054, 5.556]	0.610
Tertiary lymphoid structures (TLS) [n (%)]		
Low-grade TLS	Reference	
High-grade TLS	4.625 [1.575, 13.582]	**0.005**
Plasma exchange [n (%)]	0.785 [0.333, 1.854]	0.581

ANCA, Antineutrophil Cytoplasmic Antibody; eGFR, estimated Glomerular Filtration Rate. Bold values indicate p < 0.05.

### 3.7 Prognostic nomogram for renal survival

Based on the four independent predictors identified in the multivariate model (eGFR, complement C3, TLS grade, and Berden classification), we constructed a prognostic nomogram to predict the probability of ESRD occurrence at 1 year (**Figure 6**). The nomogram assigns a point score to each predictor, with the sum of points corresponding to the predicted ESRD probability. The C-index of the nomogram was 0.897, indicating good discriminative ability. The bootstrap-corrected calibration curve showed good agreement between the predicted and observed ESRD probabilities for both 1-year predictions (**Supplementary Figure 1**), suggesting that the model is well-calibrated.

**Figure 6:**
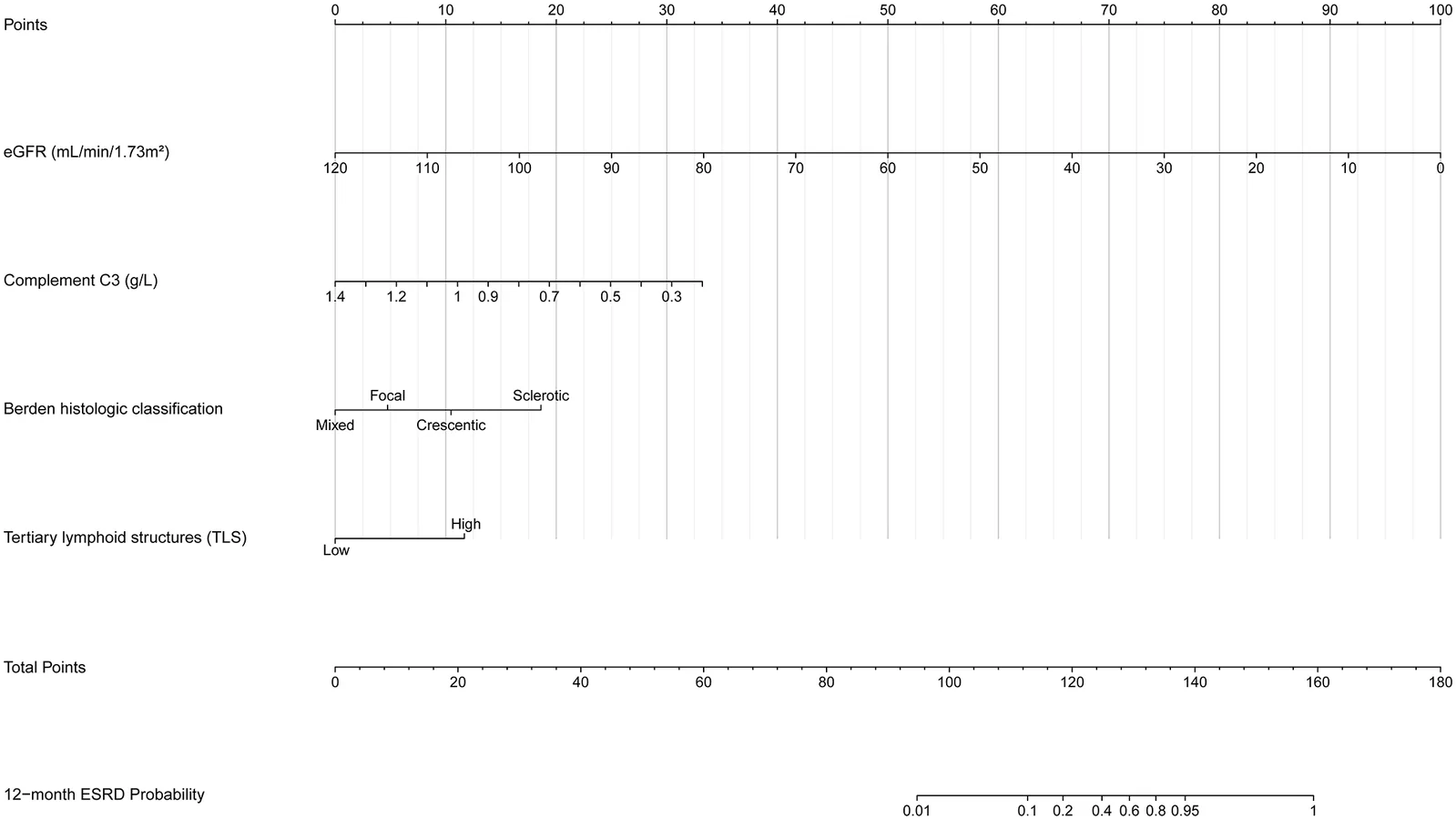
Prognostic nomogram for predicting ESRD risk in ANCA-associated glomerulonephritis. The nomogram integrates TLS grade, eGFR, complement C3, and Berden histopathological classification to estimate the probability of ESRD occurrence at 1 year.

## 4 Discussion

In this single-center, retrospective analysis of patients with ANCA-associated glomerulonephritis, we observed that individuals with high-grade TLS in their renal biopsies tended to exhibit more severe renal impairment at baseline, a higher proportion of crescentic and sclerotic histopathological classes, and a numerically greater incidence of progression to end-stage renal disease compared to those with low-grade TLS. Additionally, high-grade TLS appeared to be associated with increased intrarenal infiltration of CD20^+^ B cells and CD21^+^ follicular dendritic cells, a higher percentage of circulating CD3^+^ T cells, and an elevated Inflammation Burden Index. Critically, our multivariate analysis demonstrated that high-grade TLS was independently associated with ESRD, even after adjustment for eGFR, complement C3, and the Berden histopathological classification. Furthermore, we developed a TLS-integrated nomogram that demonstrated good discriminative ability (C-index = 0.897) and good calibration, providing a practical tool for individualized risk stratification.

The presence of organized lymphocytic infiltrates in ANCA-associated glomerulonephritis was first systematically characterized by Steinmetz et al., who identified four distinct organizational levels of intrarenal B-cell aggregates-ranging from scattered B cells (grade 1) to highly compartmentalized clusters with central CD21^+^ follicular dendritic cell networks (grade 4) (10). Subsequently, Brix et al. extended this work in a prospective cohort of 112 patients with newly diagnosed ANCA-associated glomerulonephritis and demonstrated that a higher degree of intrarenal lymphocytic organization was significantly associated with worse renal function at baseline and during follow-up, as well as a greater risk of progression to end-stage renal disease (11). Our observations align with and extend these findings by demonstrating that higher-grade TLS are not only associated with adverse renal outcomes but also correlate with specific features of local B-cell and follicular dendritic cell infiltration, as well as systemic inflammatory indices.

The cellular and molecular mechanisms underlying TLS formation and function have been increasingly elucidated in other autoimmune and chronic inflammatory conditions, offering a framework for interpreting our observations in ANCA-associated glomerulonephritis. In Sjögren’s syndrome, single-cell transcriptomic and spatial proteomic analyses have identified a specialized population of immunologically active fibroblasts, termed immunofibroblasts, that express key chemokines such as CCL19 and CCL21, thereby orchestrating lymphocyte recruitment and compartmentalization within developing TLS (12). These fibroblasts differentiate from tissue-resident progenitors in response to local inflammatory cues and establish a structural network that supports B-cell activation, germinal center formation, and sustained adaptive immune responses (12, 13). Notably, podoplanin-expressing macrophages have also been implicated in TLS formation in Sjögren’s syndrome, where they localize to the periphery of organized lymphoid aggregates and may contribute to the maintenance of these structures (13). In lupus nephritis, bioinformatic analyses have identified STAT1 and PSMB9 as potential hub genes associated with TLS development, implicating interferon signaling and antigen processing pathways in the pathogenesis of intrarenal lymphoid neogenesis (14). Furthermore, immune cell infiltration analyses in lupus nephritis have demonstrated correlations between TLS-associated gene signatures and the abundance of plasma cells, activated dendritic cells, and NK cells, reinforcing the concept that TLS serve as local hubs for antigen presentation and humoral immunity (14). In IgG4-related kidney disease, organized TLS are sustained by intricate T-follicular helper (Tfh) cell-B-cell interactions that drive local immunoglobulin production and fibrosis (15). Specifically, infiltration of Tfh cells within TLS promotes B-cell differentiation, immunoglobulin class switching, and germinal center reactions. Within the TLS microenvironment, diverse cell types contribute to fibrogenesis: Tregs produce IL-10 and TGF-β, CD4^+^ cytotoxic T cells express TGF-β and proinflammatory cytokines such as IFN-γ, and M2 macrophages secrete CCL18 to promote collagen deposition, collectively sustaining local fibrosis and tissue injury (15). Extrapolating from these findings, the high-grade TLS observed in our ANCA-associated glomerulonephritis cohort may similarly reflect a self-amplifying cycle of local chemokine production, fibroblast activation, and lymphocyte recruitment that perpetuates renal inflammation independently of systemic disease activity. This concept is further supported by recent experimental evidence from aged injured kidneys, in which single-nucleus RNA sequencing and functional validation demonstrated that TLS function as “inflammation amplifiers”: lymphocytes within TLS promote proinflammatory phenotypes in surrounding proximal tubular cells and fibroblasts via TNFα and IFNγ production, and these proinflammatory parenchymal cells in turn recruit and activate additional immune cells through chemokine secretion, thereby establishing a bidirectional feed-forward loop that sustains tissue inflammation and drives progressive fibrosis (16). The selective enrichment of CD20^+^ B cells and CD21^+^ follicular dendritic cells within high-grade TLS in our study is consistent with the notion that these structures support local antigen-driven B-cell responses, which could theoretically contribute to the persistence of renal injury even after the initiation of immunosuppressive therapy. However, the precise cellular sources of TLS-organizing chemokines and the functional competence of intrarenal germinal centers in ANCA-associated glomerulonephritis remain to be defined through dedicated mechanistic studies.

The selective enrichment of CD20^+^ B cells and CD21^+^ follicular dendritic cells in high-grade TLS aligns with the established view that mature TLS serve as *in situ* sites for B-cell activation and local antibody production (17, 18). This interpretation is supported by the observation that TLS in both mouse and human kidneys progress through distinct developmental stages—from simple lymphocyte aggregates to organized structures containing follicular dendritic cell networks, and ultimately to mature forms harboring germinal centers—and that the stage of TLS organization correlates with the severity of local tissue injury and inflammation (19).

Whether high-grade TLS represent an active pathogenic mechanism or merely a histological footprint of chronic irreversible renal injury warrants careful consideration. Several experimental observations argue against a purely passive role. In murine models, TLS are not static structures: they can regress upon dexamethasone treatment, with concomitant improvement in renal function, inflammation, and fibrosis, indicating that they remain dynamically responsive to therapeutic intervention (19). Single-nucleus RNA-seq evidence supports TLS as local inflammation amplifiers via a TNFα/IFNγ-driven feed-forward loop that sustains inflammation and promotes fibrosis (16). Furthermore, targeted disruption of TLS formation through ICOS blockade, which depletes follicular helper T cells, attenuates both TLS maturation and renal fibrosis, supporting a causal contribution rather than a bystander effect (20). We acknowledge the contrast between TLS regression under dexamethasone in mice and the worse outcomes in high-grade TLS patients despite similar corticosteroid exposure—likely reflecting that TLS are established at diagnosis and sustain local inflammation through mechanisms less amenable to systemic corticosteroids alone.

The correlation between high-grade TLS and lower serum C3 levels in our cohort raises the possibility that these structures may contribute to local complement activation and tissue injury. This association aligns with a growing body of evidence linking low C3 levels to adverse outcomes in ANCA-associated glomerulonephritis. Augusto et al. demonstrated that lower serum C3 levels at diagnosis correlated with a higher percentage of cellular crescents and poorer patient and renal survival (21). Similarly, Scurt et al., in a larger cohort of 164 patients, identified low C3 as the strongest negative predictor of death or ESRD at one year, particularly when C3 levels fell below 0.9 g/L (22). Tampe et al. further showed that low C3c levels were associated with higher ANCA renal risk scores and early requirement of kidney replacement therapy (23), while Brilland et al. confirmed that patients with lower C3 levels exhibited more crescentic lesions and worse renal prognosis, suggesting that these patients may represent a distinct subset with heightened intrarenal complement activation who could potentially benefit from anti-C5aR therapy (24). Collectively, these observations indicate that low C3 identifies a high-risk subgroup of ANCA-associated glomerulonephritis patients. The convergence of low C3 with high-grade TLS in our cohort raises the intriguing hypothesis that TLS may serve as local microenvironments that sustain complement-driven injury. Direct demonstration of this hypothesis would require further studies, such as complement component staining (e.g., C3d, C5aR1, or C5b-9) co-localized with CD20+ B-cell hubs on biopsy tissue, or measurement of serum C3/C3a or C5/C5a ratios. From a therapeutic perspective, this association also raises the possibility that patients with high-grade TLS might preferentially benefit from complement-targeted therapies, such as C5aR blockade with avacopan.

Our finding that baseline ANCA titers were not associated with ESRD does not exclude a pathogenic role for ANCA, but rather highlights the distinction between static measurements and dynamic antibody evolution. Samoreau et al. demonstrated in a multicenter cohort of 134 ANCA-GN patients that ANCA kinetics—specifically, a recurrent/persistent profile—correlated with renal outcomes, whereas baseline titers alone did not (25). These observations suggest that longitudinal ANCA monitoring may provide more clinically relevant prognostic information than a single baseline measurement, and that future studies incorporating serial ANCA determinations into TLS-based models may help clarify the interplay between local lymphoid neogenesis and systemic autoantibody dynamics.

The association between high-grade TLS and adverse outcomes suggests that TLS may guide B-cell-targeted therapy selection in AAV. Although rituximab is standard, it spares CD20-negative plasmablasts and tissue-resident B cells; next-generation anti-CD20 (e.g., obinutuzumab) and anti-CD19 agents (e.g., inebilizumab), which achieve deeper tissue depletion, are under evaluation and may more effectively eradicate TLS-resident autoreactive clones (26). We acknowledge that we did not formally test TLS-grade×rituximab interaction due to limited sample size and events. Although univariate analysis showed a non-significant protective trend for rituximab overall, whether high-grade TLS identifies patients with enhanced B-cell depletion responsiveness requires investigation in larger cohorts. Given TLS-driven fibrogenesis via T cell–macrophage crosstalk, anti-fibrotic strategies such as claudin-1 targeting or pirfenidone could mitigate chronic damage (26). Although speculative, these considerations underscore TLS grading as a potential tool for future trial design and patient stratification in ANCA-associated glomerulonephritis.

The potential influence of treatment regimens on the observed associations merits consideration. Although the proportions of patients receiving rituximab, cyclophosphamide, and plasma exchange were balanced between TLS groups in this cohort, detailed treatment data—including dosing protocols, cumulative doses, and treatment timing—were not systematically captured, precluding formal adjustment in the prognostic models. Moreover, different immunosuppressive agents may differentially affect TLS biology. Rituximab induces profound B-cell depletion, and given that high-grade TLS are characterized by organized CD20^+^ B-cell aggregates, rituximab might theoretically disrupt TLS integrity. Indeed, the RAVE and RITUXVAS trials demonstrated that relapses after rituximab occur predominantly upon B-cell reconstitution (27, 28), suggesting that B-cell targeting could modulate TLS-associated immune niches. However, whether specific agents differentially promote TLS regression or persistence remains unknown, and our study was not powered to formally test treatment-by-TLS interactions. Future studies with standardized treatment protocols and detailed medication records are needed to clarify these relationships.

Beyond its prognostic implications, the clinical applicability of TLS grading warrants consideration. Our grading system, ranging from G0 to G4 based on lymphoid organization and CD21^+^ follicular dendritic cell networks, requires immunohistochemical staining for CD3, CD20, and CD21 in addition to routine H&E evaluation, which may limit its immediate feasibility in routine practice. Moreover, although immunohistochemistry-based TLS assessment likely achieves better interobserver agreement than H&E alone, interobserver variability has not been formally assessed for this specific scheme in ANCA-associated glomerulonephritis. Standardized training protocols, consensus reading sessions, and digital pathology approaches will be essential to establish the reproducibility and practical utility of TLS grading before clinical implementation.

Several limitations should be noted. The single-center, retrospective design and limited sample size constrain the statistical power for robust multivariate adjustment. In the multivariate Cox model, the events-per-variable ratio was approximately 5.8, raising the possibility of model overfitting. This limited statistical power is reflected in the particularly wide confidence intervals observed for complement C3 and sclerotic Berden class, indicating that these point estimates should be interpreted as preliminary and unstable rather than precise. Additionally, the mixed Berden class included only 6 patients, likely explaining its counterintuitive lower point score than focal class in the nomogram. Although the mixed class was more frequent in the low-grade TLS group, this likely reflects chance given the small sample size and does not weaken the robust association between high-grade TLS and crescentic/sclerotic classes. The nomogram lacked external validation, limiting its generalizability. TLS grading may also be subject to sampling variability, and the median follow-up of 15.3 months is relatively short. Our predominantly MPO-ANCA-positive cohort may limit applicability to PR3-ANCA-positive or GPA-predominant populations. Detailed treatment dosing data were not available for granular adjustment, and functional characterization of TLS-resident cells was beyond the study scope. These findings should therefore be viewed as preliminary and hypothesis-generating.

In conclusion, this exploratory study suggests that high-grade intrarenal TLS are associated with more severe renal impairment, aggressive histology, and poorer kidney survival in ANCA-associated glomerulonephritis. High-grade TLS was identified as an independent risk factor for ESRD after adjusting for eGFR, C3, and Berden classification, and a TLS-integrated nomogram with excellent discriminative ability was developed for individualized risk prediction. While TLS grading may hold promise as an adjunctive prognostic tool, validation in larger, multicenter, prospective cohorts with multivariate adjustment is essential before clinical implementation. Future studies employing spatial transcriptomics and high-dimensional immune profiling will be needed to dissect the mechanisms driving TLS formation and to evaluate the therapeutic potential of targeting pathological lymphoid neogenesis in this disease.

## Data Availability

The raw data supporting the conclusions of this article will be made available by the authors, without undue reservation.
