# Supplementary material for: Histological grading of tertiary lymphoid structures correlates with renal impairment and adverse outcomes in ANCA-associated glomerulonephritis
Source: Front Immunol. 2026 Sep 16;17:1916074. doi: 10.3389/fimmu.2026.1916074 (PMC13624107; doi:10.3389/fimmu.2026.1916074)
Supplement: Supplementary file 1 [file DataSheet1.pdf]

Supplementary Material

1 Supplementary Tables

Supplementary Table 1. MPO-ANCA titers and ESRD risk: univariate Cox regression in the MPA subgroup

Variable	HR(95% CI)	p value
MPO-ANCA titer (RU/mL)	0.997 [0.991, 1.002]	0.204

2 Supplementary Figures

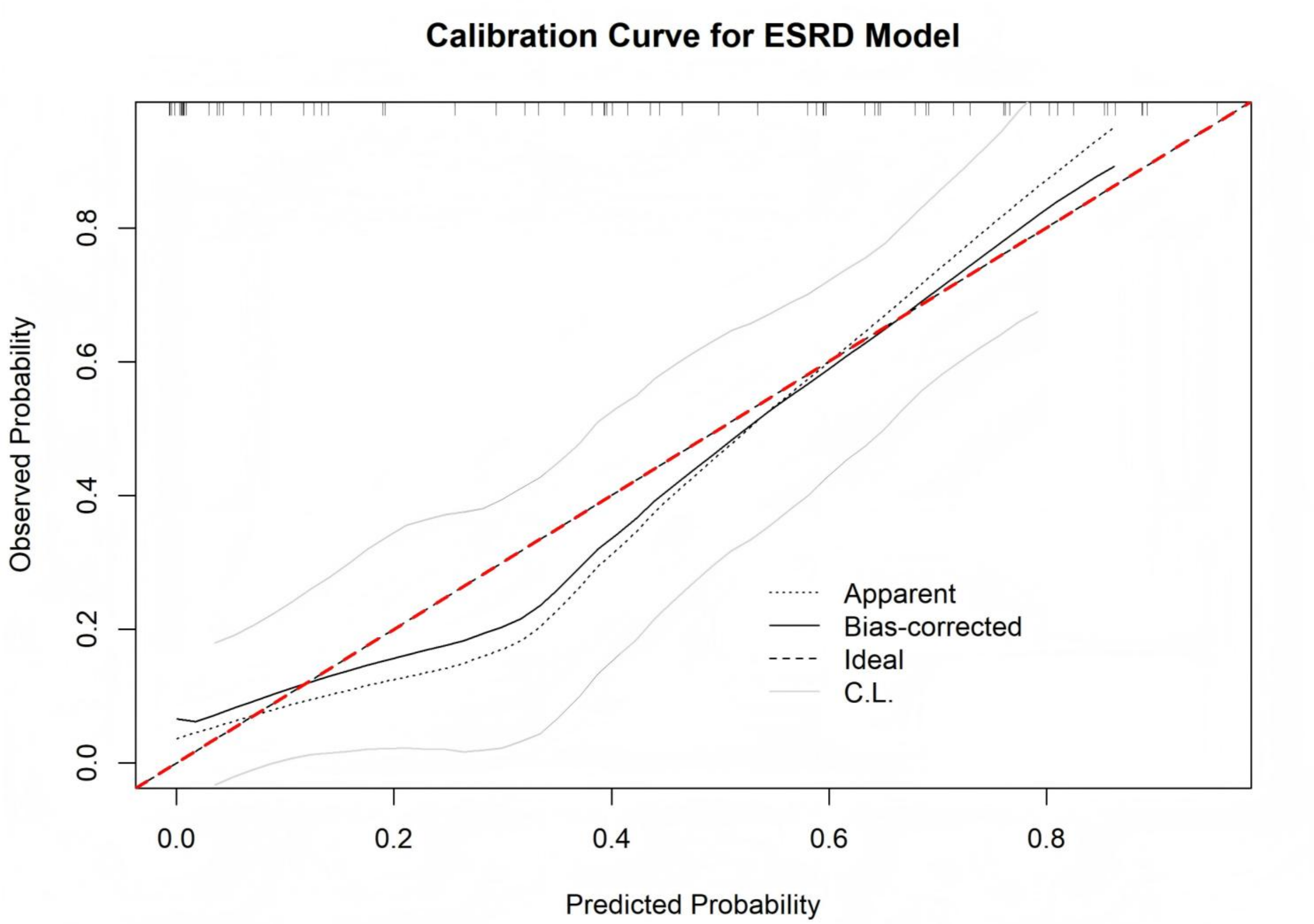

Supplemental Figure 1. Calibration curves of the nomogram model.
